# Initial dispersal behavior and survival of non-native juvenile Burmese pythons (*Python bivittatus*) in South Florida

**DOI:** 10.1186/s40850-021-00098-2

**Published:** 2021-12-08

**Authors:** Shannon E. Pittman, Ian A. Bartoszek

**Affiliations:** 1 College of Arts and Sciences, Athens State University, S 303 C, Waters Hall, Athens, Alabama 35611 USA; 2 Biology Department, Davidson College, Davidson, NC USA; 3 Conservancy of Southwest Florida, Naples, FL 34102 USA

**Keywords:** Burmese python, Dispersal, Movement, Invasive species, Management

## Abstract

**Background:**

Dispersal behavior is a critical component of invasive species dynamics, impacting both spatial spread and population density. In South Florida, Burmese pythons (*Python bivittatus*) are an invasive species that disrupt ecosystems and have the potential to expand their range northward. Control of python populations is limited by a lack of information on movement behavior and vital rates, especially within the younger age classes. We radio-tracked 28 Burmese pythons from hatching until natural mortality for approximately 3 years. Pythons were chosen from 4 clutches deposited by adult females in 4 different habitats: forested wetland, urban interface, upland pine, and agricultural interface.

**Results:**

Known-fate survival estimate was 35.7% (95% CI = 18% - 53%) in the first 6 months, and only 2 snakes survived 3 years post hatching. Snakes moving through ‘natural’ habitats had higher survival than snakes dispersing through ‘modified’ habitats in the first 6- months post-hatching. Predation was the most common source of mortality. Snakes from the agricultural interface utilized canals and displayed the largest net movements.

**Conclusions:**

Our results suggest that pythons may have lower survival if clutches are deposited in or near urbanized areas. Alternatively, juvenile pythons could quickly disperse to new locations by utilizing canals that facilitate linear movement. This study provides critical information about behavioral and life history characteristics of juvenile Burmese pythons that will inform management practices.

**Supplementary Information:**

The online version contains supplementary material available at 10.1186/s40850-021-00098-2.

## Background

Dispersal is a critical determinant of species’ spatial ecology and is particularly important in the population dynamics and spread of invasive species [1–3]. The range expansion of invasive species is often influenced by the behavior of individuals at the outer edges of the established population. Incursions into new areas are oftentimes by dispersing juveniles [2]. Prevention of further invasion is often more effective and less costly than control after establishment. Therefore, if dispersal of invasive species can be predicted, this information could manifest in a critical control tool for natural resource managers [4–7]. Many models of invasive spread assume random movement through homogenous landscapes [8, 9]. However, these assumptions are rarely upheld. Habitat-specific movement and survival during the dispersal life stage can heavily impact the spatiotemporal dynamics of population expansion, with important implications for control efforts and natural resource management [10–12].

The Burmese python (*Python bivittatus*) is an invasive species in the unique ecosystems of South Florida, USA. Native to southeastern Asia, Burmese pythons were introduced into southern Florida via the release of unwanted pets [13]. They were first recognized as established in Everglades National Park in 2000 and have established over 8,000 km^2^ in South Florida, including all of Everglades National Park and much of Big Cypress National Preserve. Burmese pythons exhibit strong negative impacts on native mammal and bird populations [14–17] and indirect effects on non-prey species [18]. A study of adult Burmese python movement in their native range found that pythons did not avoid human-dominated landscapes [19], suggesting that human development may not impede population spread in their invasive range. Rodda et al. 2011 [20] suggested that Burmese pythons could expand their range north of South Florida. Understanding habitat features that facilitate or limit dispersal, as well as elements that carry higher or lower risks of juvenile mortality, are important for identifying potential invasion corridors, or habitat types that should be the focus of management efforts [11].

The development of effective python control methods is critical to controlling impacts and limiting range expansion. Developing and evaluating control methods for Burmese pythons is particularly difficult given the low detectability of the species in the wild [21, 22]. Low detectability also hinders estimation of vital rates and population density. Radio-telemetric studies and presence-only models have provided important insight into adult Burmese python habitat use and relative habitat suitability throughout Florida [23–26]. Currently the most widely used methods of population control are direct removal from opportunistic searches in accessible habitats (such as roads), high use habitats (such as tree islands), or removal during searches using scout snakes [23, 27]. However, estimating the effects of these removal efforts on the invasion dynamics of Burmese pythons is limited by a lack of information on the behavioral ecology and survival of the younger age classes.

Our study focused on the initial dispersal behavior and survival of free-ranging juvenile Burmese pythons in heterogeneous and human-dominated landscapes in South Florida. We aimed to quantify net movement rates, habitat boundary behavior, growth, and survival over 3 years. We predicted that juveniles would display high net movement rates in natural habitat, would prefer natural habitat over human-altered habitat, and would have higher survivorship and growth rates in natural habitat than human-altered habitat.

## Results

### Study subjects

Mean mass of neonatal snakes included in this study was 136g (range = 130g – 141g) for the FW clutch, 114g (range = 92g – 127g) for the U clutch, 167g (range = 162g – 176g) for the AF clutch, and 126g (range = 124g – 128g) for the UP clutch. Mass differed significantly among the 4 clutches (Kruskal-Wallis X^2^ = 24.4, df = 3, *p*<0.001). Post-hoc tests indicated significant differences in mass between AF and UP (*p*<0.01), AF and U (*p*<0.001), and FW and U (*p*<0.05). Maternal mass, maternal SVL, and maternal total length for each clutch was as follows: 47.2 kg, 376 cm, and 427 cm for FW, 25.9 kg, 300 cm, and 341 cm for U, 83.9 kg, 431 cm, and 490 cm for AF, and 30.1 kg, 330 cm, and 377 cm for UP.

### Survival

**Fig. 1:**
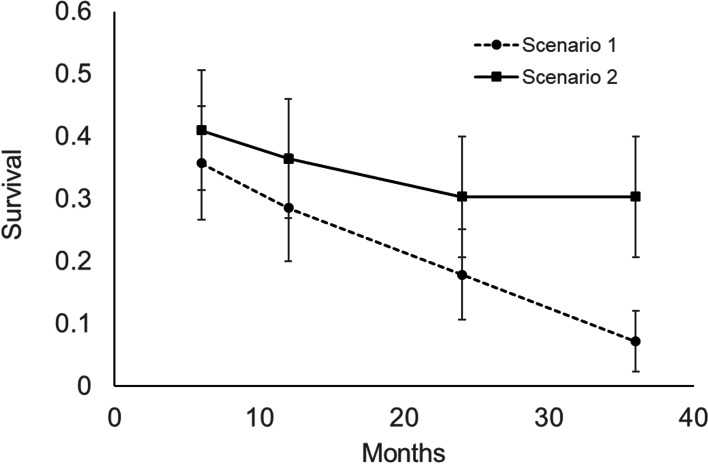
Mean survival (+/- SE) of 28 pythons over 3 years using known-fate analysis. The dashed line represents estimates from Scenario 1, where unrecovered pythons were presumed deceased, while the solid line shows estimates from Scenario 2, where unrecovered pythons were censored in the known-fate analysis

**Fig. 2:**
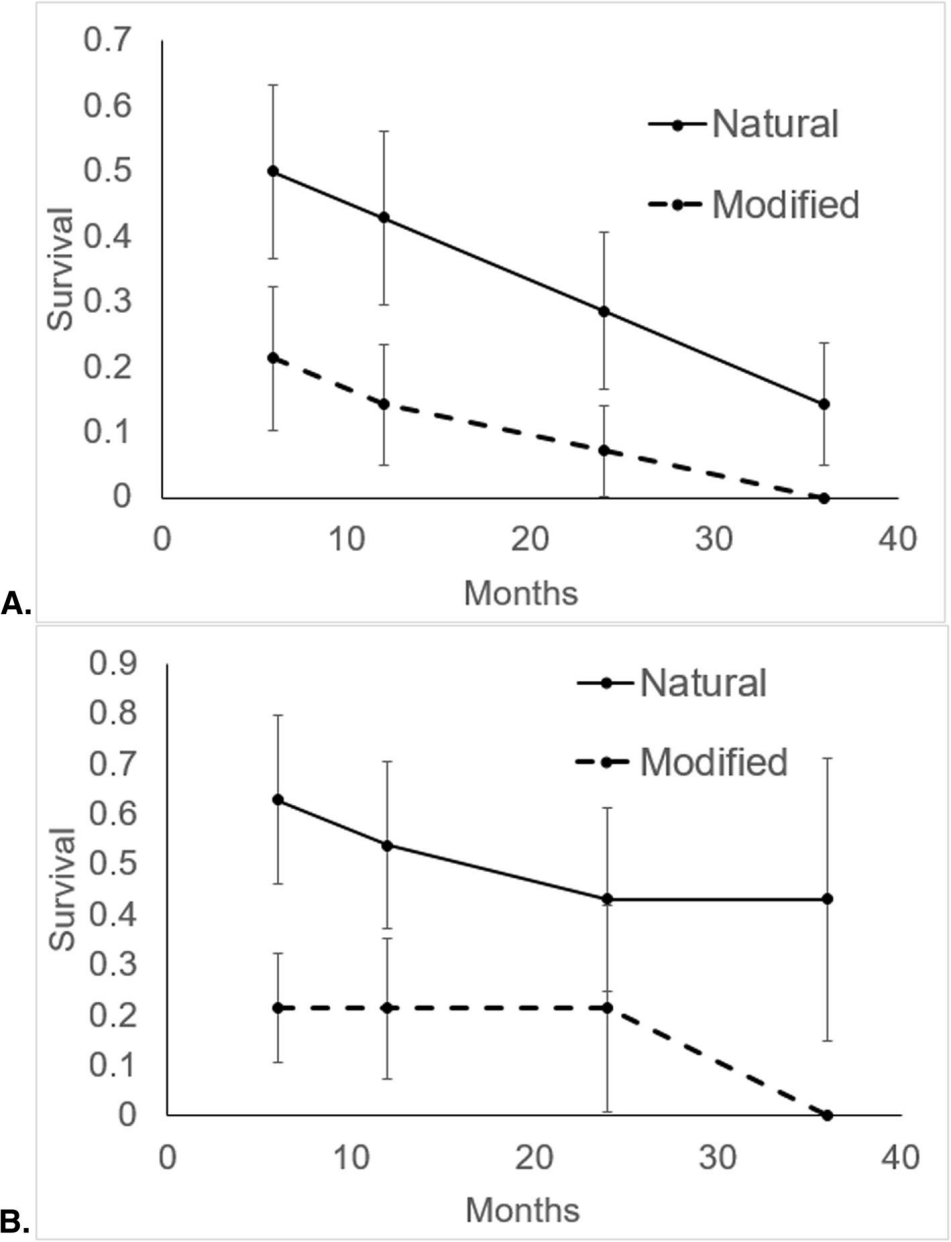
Mean survival (+/- SE) of pythons over 3 years separated by habitat type, where ‘modified’ refers to U and AF and ‘natural’ represents FW and UP. Part A represents estimates from Scenario 1, where unrecovered pythons were presumed deceased, while Part B shows estimates from Scenario 2, where unrecovered pythons were censored in the known-fate analysis

The known-fate estimate of 6-month survivorship of neonates was 35.7% (SE = 0.090; 95% CI = 18–53%) for Scenario 1 and 41% (SE = 0.096; 95% CI = 21–60%) for Scenario 2 (Fig. 1). First year survivorship was 28.6% (SE = 0.085; 95% CI = 12-45%) for Scenario 1 and 36.4% (SE = 0.095; 95% CI = 16–57%) for Scenario 2. Only 2 out of 28 snakes were confirmed to have survived 3 years post release (7.1%). Of those that were confirmed to have survived for at least 6 months (those not deceased or censored), 4 were FW, 3 were UP, 2 were U, and 1 was AF. For Scenario 2, we found that 6-month survival of those released in ‘natural’ habitat (FW or UP) was higher than survival of snakes released into ‘modified’ habitat (U or AF; Χ^2^= 3.93, df = 1, *p* = 0.047; Fig. 2). However, this difference in survivorship was non-significant at 12 months (Scenario 1: Χ^2^= 3.13, df = 1, *p* = 0.076; Scenario 2: Χ^2^= 2.07, df = 1, *p* = 0.15; Fig. 2). We did not find a significant difference in survivorship between neonates released in 2014 versus 2015 (Scenario 1: Χ^2^= 1.03, df = 1, *p* = 0.31; Scenario 2: Χ^2^= 0.52, df = 1, *p* = 0.47).

Documented sources of mortality were predation (*n*=12; mammal, alligator, indigo snake [28]), starvation (*n*=1), injury (*n*=1), and unknown (*n*=3). Eight snakes were unable to be located and transmitters were never recovered (these were ‘censored’ in Scenario 2). The majority (~70%) of confirmed mortality events were attributable to predation – including 3 instances of radio-transmitters tracked to an alligator (indicative of the radio-transmitter in the alligator’s digestive tract). We did not detect a difference in sources of mortality among our 4 clutches.

### Growth

**Fig. 3:**
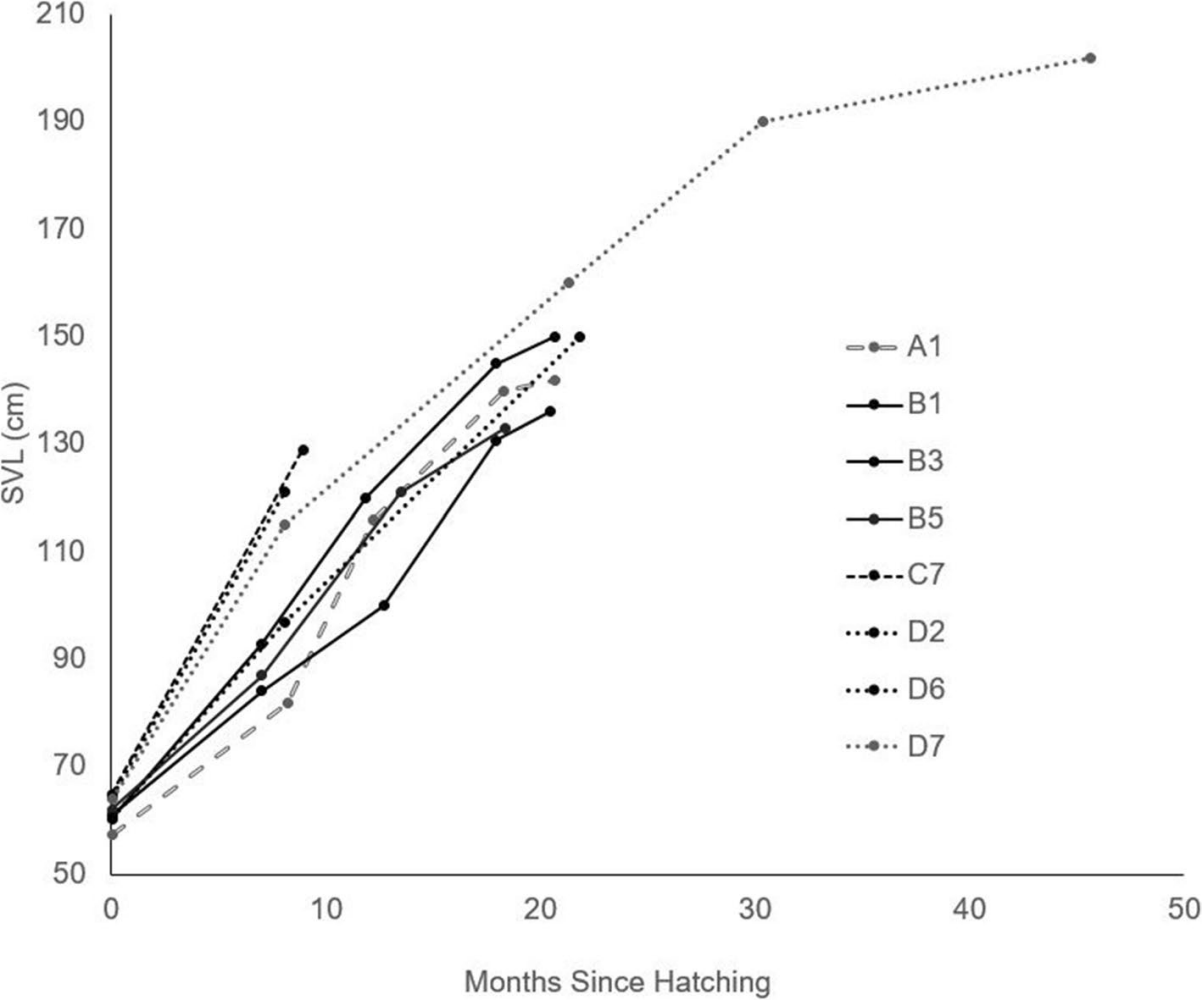
Snout to vent length (SVL) of recaptured snakes. Boxed line represents snake released in U. Solid lines represent snakes released into FW. Dashed line represents snake released in AF. Dotted lines represent snakes released in UP

Of the 28 snakes within the study, 8 survived to be recaptured and measured at least once. Linear growth rates between successive recaptures peaked within the first ~12-14 months after hatching (5 out of 6 snakes that were measured more than once) (Fig. 3). Because of the small sample size of recaptured snakes, we were not able to compare linear growth rates among clutches. Four snakes were recaptured and measured between 11 and 13 months after release (i.e. within 1 month of a year), and the mean growth rate was 4.32 cm/day (SD = 0.88, range = 3.07 – 5.03). Mean growth rates between 8 – 14 months (the recapture as close to 1 year as possible for each recaptured snake) were 4.16 cm SVL/month for FW (*n*=3; SD = 0.99), 5.93 cm SVL/month for UP (*n*=3; SD = 1.27), 4.82 cm SVL/month for U (*n*=1), and 7.2 cm SVL/month for AF (*n*=1). We found no significant difference in growth rates between males and females (W = 10, *p* = 0.5714). Notably, the one snake measured from AF (C7) reached 184 cm SVL (209 cm total length, 3.35kg) after 397 days (Fig. 3). One snake (D7) in this study was recovered and measured after 45 months. This snake was included in Fig. 3 to show the growth curve beginning to asymptote.

### Movement behavior

**Fig. 4:**
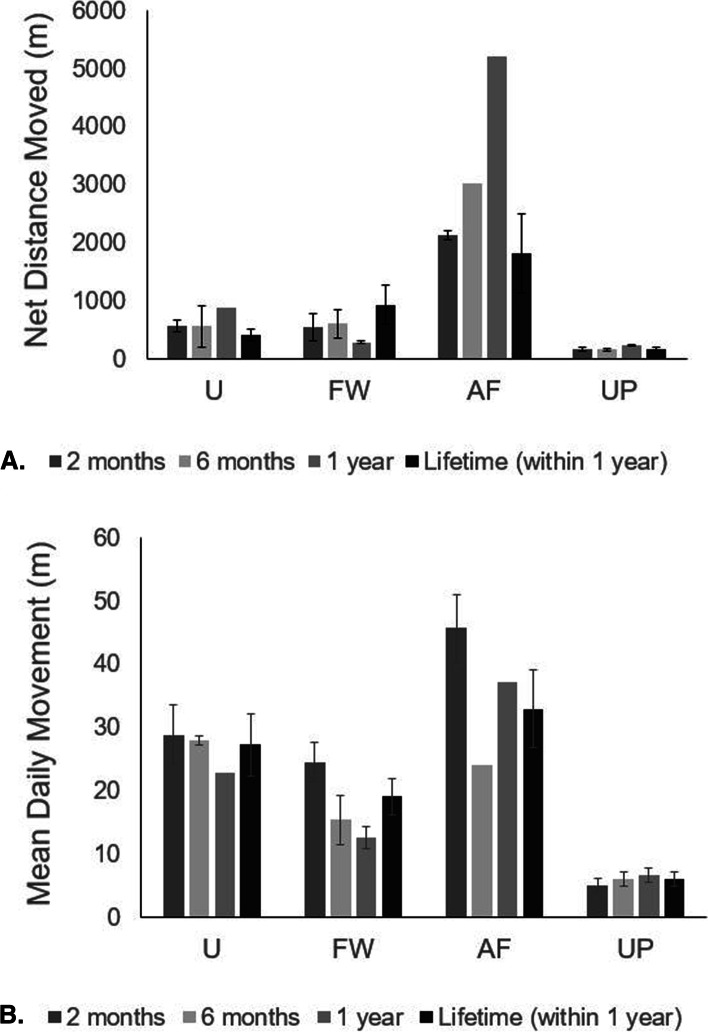
Mean (+/- SE) net movement (**a**) and daily movement (**b**) of snakes released at each of the four habitat locations – Urbanized (U), Forested Wetland (FW), Agricultural Field (AF), Upland Pine (UP) at 2 months, 6 months, 1 year, and lifetime (within 1 year, including deceased snakes). High movement rates for the AF clutch may reflect extensive canal use

The four clutches differed significantly in mean distance moved per day and net distance moved after 2 months (mean distance: F = 14.9, df = 3,19, *p* < 0.001; net distance: F = 18.33, df = 3,19, *p* < 0.001). Sample sizes became too small for inferential tests at the 1-year mark, but there were large differences in movement between the remaining snakes among habitats (Fig. 4). Post hoc tests indicated differences among clutches in net distance traveled were driven by the high net movements of AF snakes (AF clutch differed significantly from all other clutches; Fig. 4; Supplementary Table 1).

**Fig. 5:**
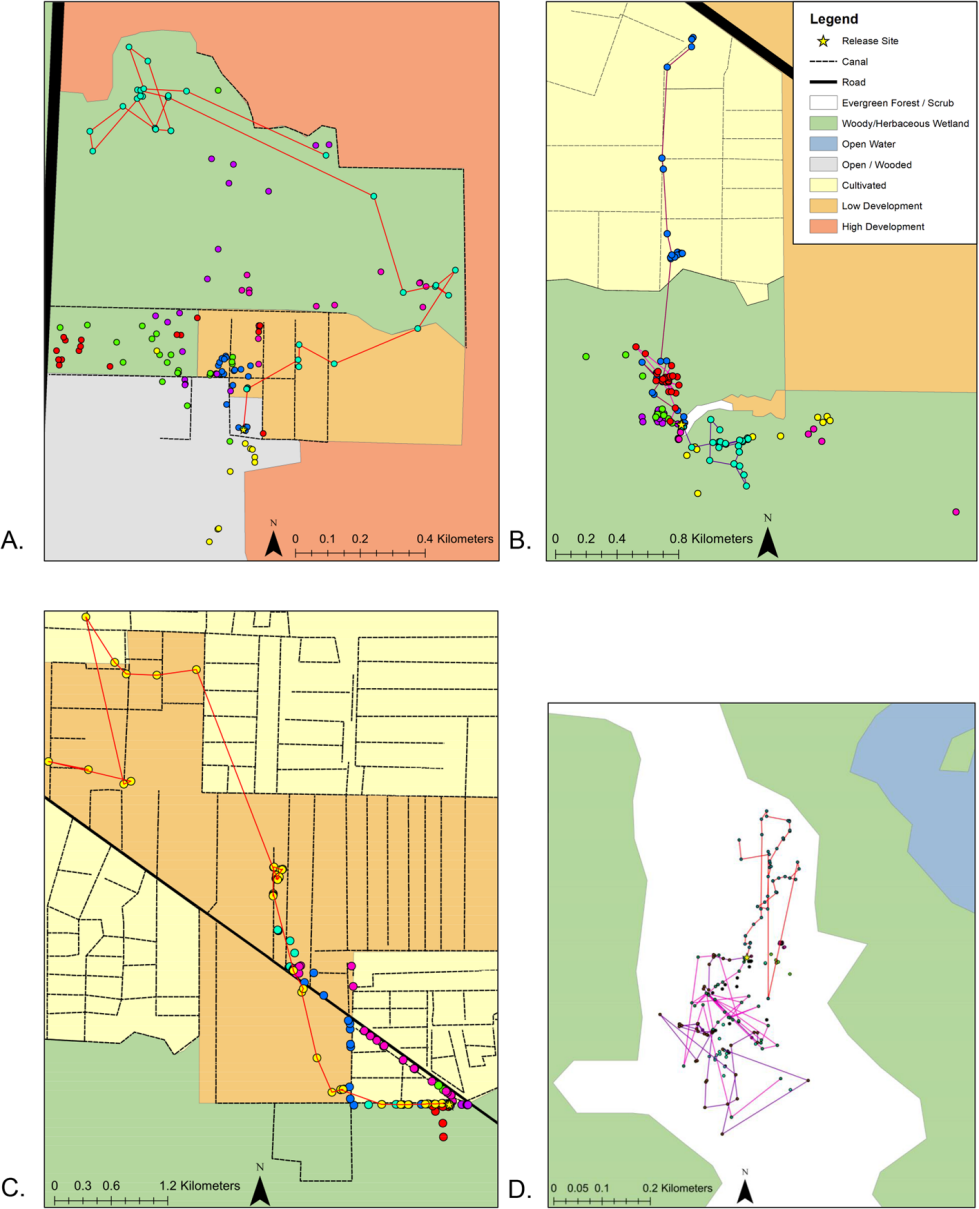
One-year movement of the 28 snakes released in each habitat. **A** refers to U habitat, **B** refers to FW habitat, **C** refers to AF habitat, and **D** refers to UP habitat. Each color represents a different snake and each dot represents one location. Snakes with 1 full year of movement data (survived the full year) have lines connecting locations. The star represents the clutch site and release location. Low traffic neighborhood roads or gravel agricultural roads are not represented on the map. Note the difference in spatial scales represented in **A**-**D**

The highest net movement rates occurred for the AF clutch, with one snake (C7) traveling a net distance of 6448m within 365 days (Table 1). Notably, all 7 snakes in the AF clutch were located in or near canals at least once after release (Fig. 5C). Four out of 7 snakes in the AF clutch crossed a 2-lane road and traveled through low intensity developed habitat (Fig. 5C). Although FW snakes were not released in close proximity to canals, the one snake (B3) that moved far enough north to encounter canals used the canals extensively (Fig. 5B). UP snakes displayed the smallest net distances moved, with a mean net distance traveled of 234m at 1 year (*n*=3; Fig. 5D).

**Table 1:** Individual survival and movement data for 28 Burmese pythons. Maximum observed net distance moved refers to the maximum distance a snake was observed to have moved from the release location within 1 and 2 years. Snakes that did not survive longer than 1 year were given the ‘n/a’ (not applicable) designation in the 2 year column

ID	Release habitat	Sex	Release Date	# Days confirmed alive (up to 3 years or 1095 days)	Max net distance within 1 year (meters)	Max net distance within 2 years (meters)
A1	U	M	7/22/2014	951	654	654
A2	U	F	7/22/2014	255	1049	n/a
A3	U	F	7/22/2014	125	233	n/a
A4	U	M	7/22/2014	68	715	n/a
A5	U	F	7/22/2014	79	626	n/a
A6	U	M	7/22/2014	94	917	n/a
A7	U	F	7/22/2014	70	362	n/a
B1	FW	M	7/22/2014	717	585	918
B2	FW	M	7/22/2014	70	761	n/a
B3	FW	M	7/22/2014	1095	2517	2898
B4	FW	F	7/22/2014	56	1875	n/a
B5	FW	F	7/22/2014	756	583	823
B6	FW	F	7/22/2014	185	260	n/a
B7	FW	F	7/22/2014	89	966	n/a
C1	AF	M	7/15/2015	106	2595	n/a
C2	AF	M	7/15/2015	38	231	n/a
C3	AF	M	7/15/2015	51	2006	n/a
C4	AF	M	7/15/2015	99	2191	n/a
C5	AF	F	7/15/2015	17	356	n/a
C6	AF	F	7/15/2015	15	197	n/a
C7	AF	F	7/15/2015	397	6448	6448
D1	UP	F	7/15/2015	79	210	n/a
D2	UP	F	7/15/2015	543	261	720
D3	UP	F	7/15/2015	79	290	n/a
D4	UP	F	7/15/2015	26	96	n/a
D5	UP	M	7/15/2015	59	81	n/a
D6	UP	M	7/15/2015	417	371	371
D7	UP	M	7/15/2015	1095	306	1036

The initial movement bearings of snakes released in natural habitat (FW and UP) were not significantly different from random (FW: *R* = 0.27, *p* = 0.61; UP: *R* = 0.27, *p* = 0.63). The initial movement bearings of snakes released in U were significantly different from random (*R* = 0.65, *p* = 0.045), and the V-test showed significant movement north of the clutch site in the direction of canal systems and wetland habitat (*R* = 0.60, *p* = 0.01). For snakes released in habitat type AF, Rayleigh’s test showed that snakes chose initial movement orientations randomly (*R* = 0.59, *p* = 0.086) when mean bearing was not specified; however, the V-test showed significant orientation towards eastward agricultural canals 10 days post release (*R* = 0.53, *p* = 0.023) when specifying mean direction toward the agricultural levees (Fig. 5C). Against our predictions, the V-test showed that AF snakes did not show significant orientation south toward forested wetland habitat and away from human-modified areas (*R* = -0.24, *p* = 0.82).

Habitat boundary behavior analysis (Monte Carlo tests) indicated sample sizes (number of locations per snake) were too small to detect avoidance of high-intensity developed habitats in 6 out of 7 snakes released in U. This occurred when a majority of simulated snakes did not cross into high-intensity developed habitats, and therefore avoidance behavior for the observed snakes could not be detected. For one snake (A1), 930 out of 1000 simulated movement paths crossed into high-intensity developed habitats, while A1 did not cross into high-intensity urbanized habitat (*p*=0.07). Although U snakes moved between wetland and low intensity developed habitat, none of the seven snakes in the U clutch was observed to have crossed the 4-lane road to the west of the release site (Fig. 5A) or was located in high intensity developed habitat (although we may not have detected forays into this habitat). These results could suggest that certain habitats may act as movement barriers for juvenile snakes.

## Discussion

Our results suggest that local populations of Burmese pythons may have low survival, and thus recruitment, from clutches deposited in or near urbanized areas. However, our results also suggest that juveniles may use canal systems to disperse through unfavorable habitat. AF snakes moved greater net distances than snakes released in other locations, and their movements may have been facilitated by extensive use of canal systems. U snakes seemed to avoid high-intensity urbanized habitat and also initially oriented their movements in the direction of canals and wetland areas. These results suggest that populations could expand to new locations through utilization of canals by juveniles. However, since snakes at each release location were from separate clutches, differences in movement or survival among habitats could be attributable to genetic or developmental differences among clutches [29]. Future studies should control for clutch when investigating the influence of habitat on movement and survival by interspersing individuals from different clutches into the same range of habitat treatments.

Estimates of juvenile survival are critical for managers seeking to measure the impact of removal efforts [30]. We found that survivorship was similar to or slightly lower than has been assumed in previous population modeling efforts [13]. Snakes displayed 6-month survivorship of approximately 37 – 40%, and overall first year survivorship of 21-36%. Scenarios 1 and 2 had markedly different survivorship estimates after 6 months because radio-transmitters were not recovered in eight snakes. Estimates of survivorship in Scenario 1 decreased to 21% after 12 months and to 7% after 2 years, while Scenario 2 had large uncertainty in estimates after 6 months. We estimated higher 6-month survivorship in natural compared to modified habitat in Scenario 2. Therefore, we suggest spatially explicit population models include habitat-specific survivorship, as our research suggests the potential for large differences in survival among habitat types.

Movement differed among snakes released in different habitat types. The lowest net movement rates were documented for snakes released in upland pine habitat (UP) and the highest net movement rates were documented for snakes released in agricultural habitat (AF). The distribution of resources (such as burrows and prey) was largely homogenous in the upland pine habitat (I. Bartoszek, unpubl. data), while vegetative cover is often concentrated along levees, ditches, and canal banks in the agricultural habitat. These differences may have contributed to the differences in movement patterns we observed, although mass at hatching and genetics also differed between clutches and could have contributed to differences in movement rates. Although limited by sample size, our results also suggest that pythons may display boundary behavior by avoiding high intensity urbanized habitat in favor of wetland habitat and that pythons may favor agricultural levees over woody wetland habitat. Boundary behavior in heterogeneous landscapes can have strong influences on population dynamics and the potential for spatial spread [31–33]. Therefore, future field studies should focus on investigating juvenile python boundary behavior.

The results of this study suggest that Burmese python population dynamics and spread are likely to be influenced by landscape composition. High intensity urbanization may be a dispersal barrier to juveniles, although within Florida landscapes this may be mitigated by the presence of extensive canal systems. Juvenile pythons readily use these pathways, which facilitate linear movements and may increase net dispersal distances, thereby increasing the likelihood of established satellite populations spatially disjunct from the larger source population. Simulation models of invasive species have shown that removing satellite populations may do more to control invasive spread than removing individuals from the expanding edge of a large invasive population [34, 35]. However, the low detectability of Burmese pythons will decrease the likelihood of identifying satellite populations early in the establishment phase [22]. This may reduce the efficacy of management efforts seeking to identify new populations at the invasion front, and management resources may be more effective by reducing propagule pressure through harvesting initiatives in high density source populations. Targeted environmental DNA monitoring north of extensive canal networks may be useful in identifying potential satellite populations [36]. Spatially explicit population models that incorporate habitat-specific movement and mortality may provide insight into potential locations of small satellite populations that could be the focus of future removal efforts and also may clarify the certainty with which invasion corridors can be predicted.

## Conclusions

Our results document the first estimates of free-ranging juvenile Burmese python movement rates and survival in their invasive range. These estimates provide critical information for estimating population density and will facilitate population modeling that will inform management and control practices. First-year movement behavior provides insight into how and the degree to which juveniles influence population spread. The importance of the juvenile life stage to Burmese python population expansion depends on the degree to which juveniles make long distance exploratory movements into novel territory. Our study suggests that juveniles can make long-distance movements using canal systems, but their movements are impeded by high-intensity urbanization. Adult pythons have sophisticated navigational capacities, which may decrease the risk associated with long distance exploratory movements for the adult age class [37]. Additionally, the behavior of adult Burmese pythons in their native range suggests that their movement is not impeded by human development [19]. If adults make more exploratory, long-distance movements than juveniles, adults may be the primary drivers of population expansion. Habitats that limit juvenile movements may not impede the movements of adults; therefore, invasion corridors predicted by the behavior of juveniles may not be accurate if range expansion is driven by adult movement. Long-term studies that follow the movement of multiple age classes will provide additional insight into the age classes most likely to drive population expansion.

## Materials and methods

### Study area

**Fig. 6:**
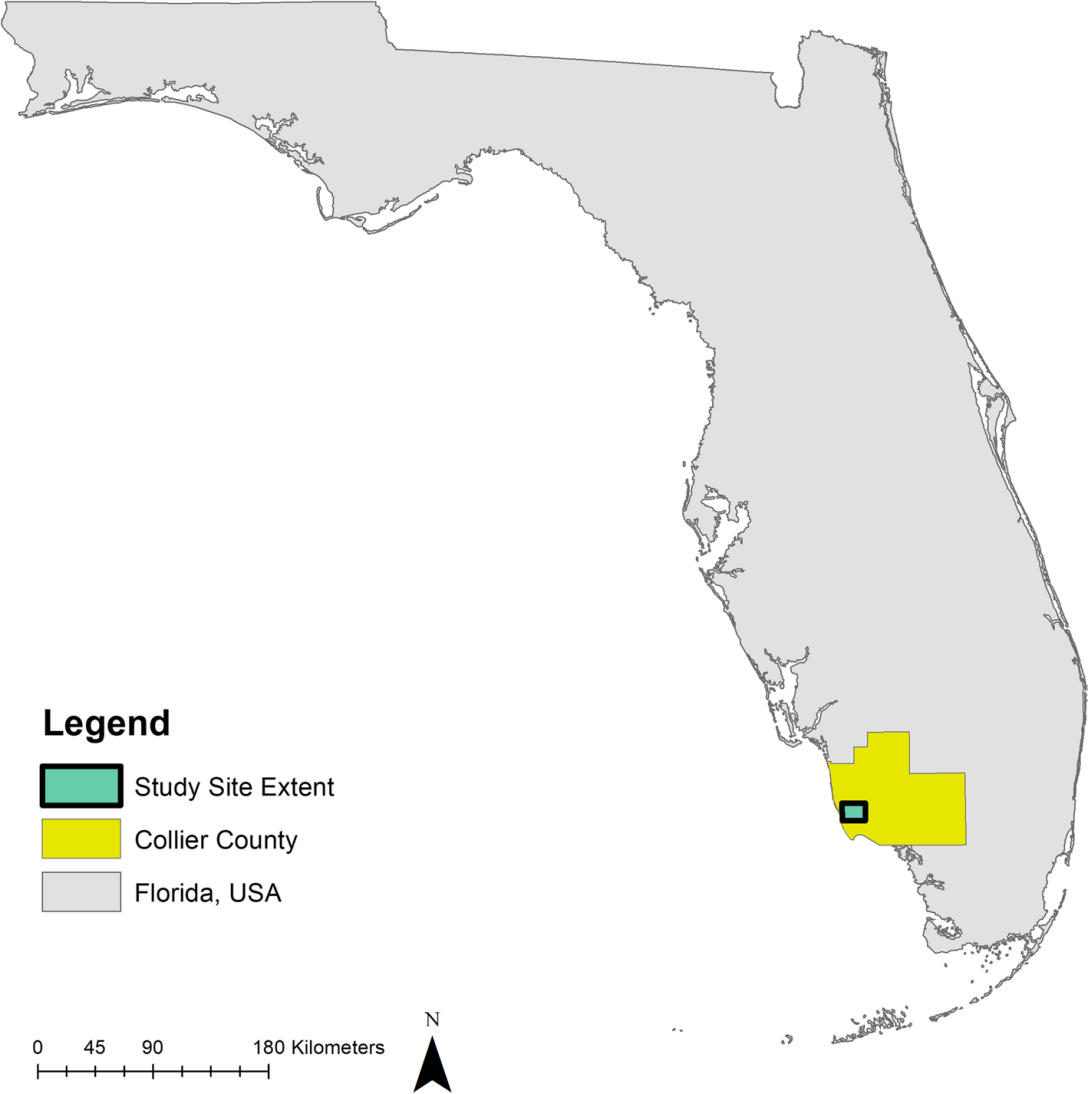
Map of Florida showing the location of Collier County and the extent of the study area. The study area extent presented is the extent of the map in Fig. 7. The state and county outlines were obtained from ESRI ArcGIS Online (Florida State Outline. ArcGIS REST Service Directory. [cited 2021 October 6] Available from: https://services1.arcgis.com/B4MnusZHL3vmqU3t/ArcGIS/rest/services/Florida_state_outline/FeatureServer/0; Collier County General. ArcGIS REST Service Directory. [cited 2021 October 6] Available from: https://services2.arcgis.com/UJQ7Q9uboSWRAzxj/arcgis/rest/services/CollierCountyGeneral/FeatureServer/0). The figure was created using ArcMap 10.3 (ESRI, Redlands, CA, USA)

**Fig. 7:**
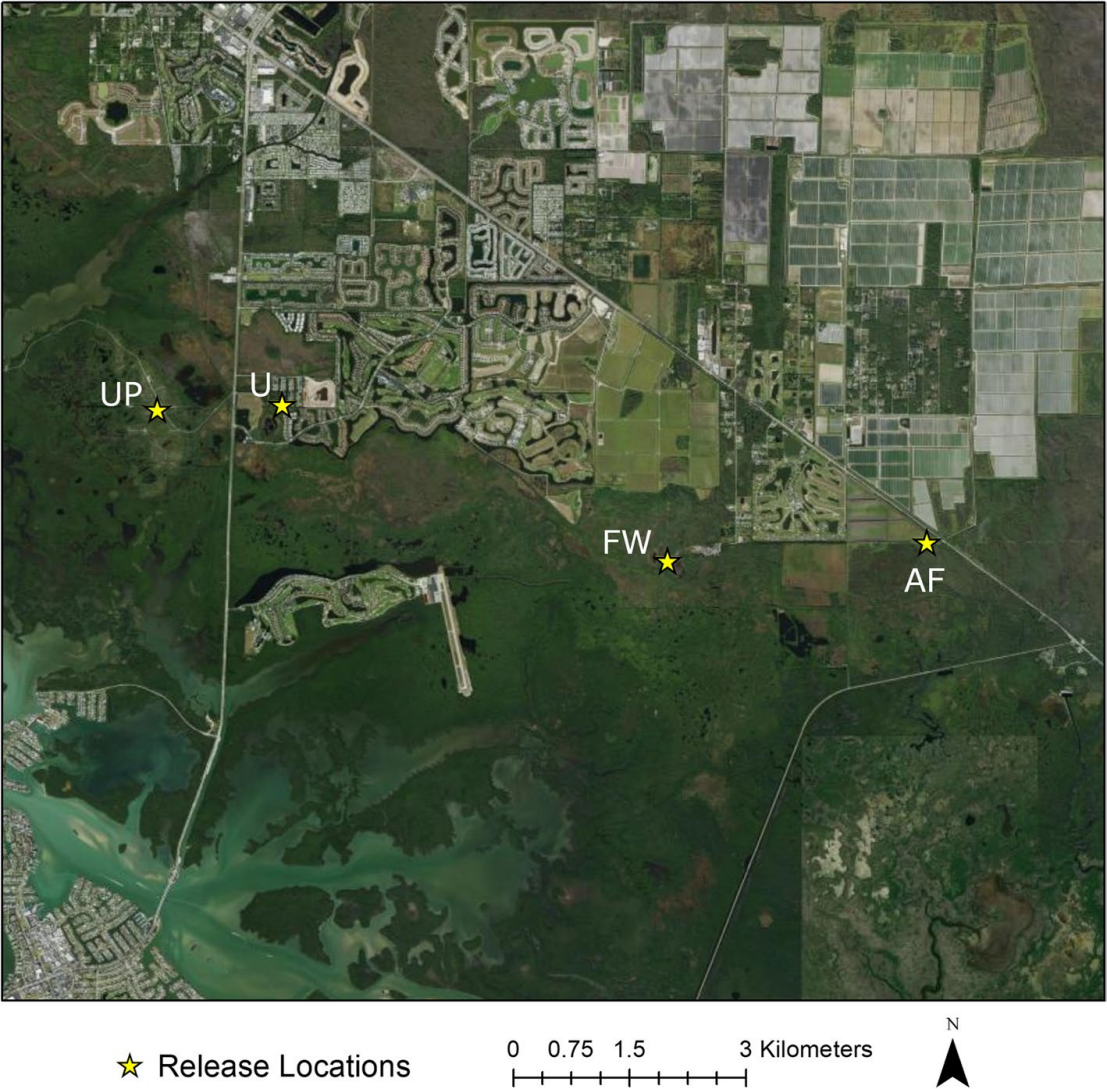
Satellite imagery map of the study area and the clutch and release site locations. The star represents the clutch site and release location. The satellite imagery was generated from the basemap “Word Imagery” (ESRI, Redlands, CA, USA; sources: from ESRI, DigitalGlobe, GeoEye, i-cubed, USDA FSA, USGS, AEX, Getmapping, Aerogrid, IGN, IGP, swisstopo, and the GIS User Community). The figure was created using ArcMap 10.3 (ESRI, Redlands, CA, USA)

All juvenile Burmese pythons used in this study were collected from Collier County, Florida, USA, within the Big Cypress Basin Watershed. The study was conducted on public lands in Rookery Bay National Estuarine Research Reserve, Collier Seminole State Park, and adjacent private lands (Figs. 6 and 7). Southwestern Florida is composed of a mosaic of habitat types including upland pine and hardwood areas, herbaceous wetlands, forested marshes, and urbanized and agricultural areas. Our study was conducted at 4 sites representing 4 distinct habitat types that are common within the region: urbanized habitat (U; Fig. 5A), forested wetland (FW; Fig. 5B), agricultural fields (AF; Fig. 5C), and upland pine (UP; Fig. 5D). The forested wetland and upland pine habitats were largely homogenous. The urban area was located near the intersection of high and low intensity development, and the agricultural area was situated at the interface of agricultural fields and forested wetland habitat (Fig. 7). The maximum straight-line distance between study sites was 10.1 km (UP and AF), and the minimum straight-line distance between study sites was 1.6 km (U and UP; Fig. 7).

### Capture and handling

We collected egg clutches in the wild deposited by female Burmese pythons being utilized in a concurrent adult radio-telemetric study [23]. We weighed and measured the female pythons in a laboratory setting one time during the breeding season, and we subsequently released the females to be radio-tracked as part of the concurrent adult movement study [23]. Clutches were incubated by the female in the wild to near full term thereby maximizing natural nest site conditions and limiting disturbance to developing eggs. Attendant female pythons were captured and removed from all clutches and released nearby the respective clutch site. Clutches were either removed from a surface nest or excavated from an underground animal burrow. The clutch was transported back to the laboratory intact and oriented in the position deposited. The final phase of incubation occurred in the lab to ensure that all hatchling snakes were collected from the clutch.

Incubation occurred inside a modified igloo 45-liter cooler (65 cm length x 37 cm width x 37 cm height). A layer of water 8 cm deep was placed on the bottom that included a submersible aquarium heater (National Geographic 5211515 aquarium heater). A grate was suspended on bricks above the water, and the clutch was placed on top of the grate. Relative humidity and temperature were monitored and kept at approximately 90% and 32°C respectively. Once the snakes emerged fully from eggshells, they were transferred to a sterile 76-liter glass aquarium with orchid bark substrate for observation prior to release.

### Radio-telemetry

We radio-tracked 28 juvenile Burmese pythons from 4 clutches (7 individuals per clutch). Fourteen juveniles (7 from each of 2 clutches (U and FW)) were released in July 2014 and 14 juveniles (7 each from 2 separate clutches (AF and UP)) were released in July 2015. All snakes included in this radio-telemetry study were released at their original nest site within 1 week of hatching. The acquisition and release of snakes in this radio-telemetry study were permitted by Florida Fish and Wildlife Conservation Commission permit number FWC EXOT-14-45. Davidson College’s Animal Care and Use Committee approved the methodology used in this study. Each clutch was the offspring of a different female snake. We did not conduct genetic analyses to determine relatedness of juveniles. Therefore, it is possible that clutches were mixed paternity or that separate clutches had the same sire [29]. The study sites ranged from 1.6 to 10 km apart, and the home range of a male python could potentially have encompassed two study sites [23, 24]. Juvenile snakes were chosen for inclusion in the study based on their masses (larger masses were preferred), sexes (3 or 4 of each sex), and coloration pattern (aberrant patterns were excluded) [38]. The seven snakes radio-tracked from each clutch consisted of either 3 males and 4 females or 3 females and 4 males (Table 1). Snakes not included in this radio-telemetry study were transferred for inclusion in separate permitted research studies. Each python was implanted intraperitoneally with a VHF radio-transmitter obtained from Holohil Systems Limited, Ontario, Canada (model SB-2, 5.0g) using the methodology described in Reinert and Cundall 1982. At the end of this radio-telemetry study, all surviving snakes were integrated into a separate adult radio-telemetry study [23].

We radio-tracked neonates using A RA-23K VHF two-element antenna (Telonics, Mesa, Arizona), and Yagi three-element antenna (Titley Scientific, Columbia, Missouri) attached to a R-1000 telemetry receiver (Communication Specialists, Incorporated, Orange, California). When a study animal was located, the UTM coordinates (NAD83; obtained with hand-held GPS unit), physical condition, and behavior at the time of observation were recorded. We made efforts to minimize disturbance to the animal. Snakes were tracked and located at least 3 times per week for the first 6 months after release. Snakes that survived beyond 6 months were implanted with a new transmitter (Holohil model SI-2, 11g) and located approximately once per month until the snake was deceased or unrecovered. Snakes were tracked visually or aerially if locations could not be ascertained via walk-in. For aerial pinpointing, we used a Cessna Skyhawk 172 aircraft with wing-mounted RA-2AHS antennae (Telonics, Mesa, Arizona). We attempted to identify predation events by determining whether the radio-transmitter was ingested by another animal (through tracking signal to another animal) or observing the type of damage to the snake or recovered radio-transmitter.

### Survival analysis

We calculated the survival of neonatal snakes using a modified Kaplan-Meier estimator [39], and we calculated 95% confidence intervals using the Greenwood formula [40]. We combined data from snakes released in 2014 and 2015 to present the 6-month, 1-year, 2-year, and 3-year survival estimates for all snakes in the study. Survival analyses were conducted for two different scenarios reflecting different assumptions regarding mortality. In both scenarios, snakes were considered ‘deceased’ if the radio-transmitter was located with signs of obvious damage or the snake itself was found dead. In Scenario 1, snakes that were unrecovered and no radio-transmitter or body was found were considered ‘deceased’ if multiple attempts to locate the animal, via both walk-in and plane, were unsuccessful over a timespan of several weeks. The type of radio-transmitters used in this study had low rates of failure (I. Bartoszek, unpublished data), and we attempted to locate all snakes repeatedly via both walk-ins and aerial overflights. Therefore, in Scenario 1, we assumed that unrecovered snakes were deceased, and radio-transmitter antennae were damaged in the mortality event.

In Scenario 2, these unrecovered snakes were ‘censored’ (removed) from the study at the time point in which they were unable to be located. We consider Scenario 1 to be a high estimate of potential mortality, and Scenario 2 is more conservative and acknowledges greater uncertainty. For both scenarios, we compared the survival estimates between those animals released in ‘natural’ (FW and UP) versus ‘modified’ habitats (U and AF), and we also compared survival between release years (2014 and 2015). For all statistical comparisons of survival point estimates, we used chi-square tests with an arcsine transformation of the survival function to account for our small sample size [40].

### Growth

Snout to vent length (SVL) and mass were measured for each snake upon hatching and when snakes were recovered for radio-transmitter replacement (every 5-10 months). We presented growth data for snakes that survived long enough to be refitted with radio-transmitters. We presented a summary of the SVL for each snake at each recovery. We compared the masses at hatching among the 4 different clutches using the Kruskal-Wallis test and performed post-hoc tests using the Dunn test. We compared linear growth rates between males and females using the Wilcoxon rank sum test. We used an alpha of 0.05 as a standard for significance.

### Movement analysis

We used ArcGIS 10.3 (ESRI, Redlands, CA, USA) to plot and summarize python movement paths. We calculated mean movement distance per day per snake and net distance moved at 2 months, 6 months, 1 year, and final net distance moved within 1 year. We calculated net distance moved by quantifying the straight-line distance between the observed location and the release site [41]. We also investigated differences in movement behavior (net distance and mean distance moved per day) among clutches using ANOVA. Mean distance moved per day was calculated by dividing the net distance moved between locations by the number of days between locations. We presented the maximum observed net distance moved after 2 years, but we do not present mean distance moved per day after 1 year because snakes were too infrequently located for this measurement to be meaningful (located only once per month).

We used ArcGIS 10.3 and R statistical software to assess behavior of individuals released on or close to habitat boundaries (applicable to U and AF clutches). We plotted python movement within different land cover categories using ArcGIS 10.3 and Land Use / Land Cover data from Florida Cooperative Land Cover Map, version 3.0 and merged additional geospatial data on canals and ditches from the South Florida Water Management District and direct observation in the field.

For all clutches, we tested the movement bearings of snakes 10-days post release (when the majority of snakes had moved at least 10 m from the clutch site) to determine whether the initial movement from the clutch site was significantly different from random. We predicted that snakes released in homogenous habitat would disperse randomly from the clutch site (FW and UP), while we predicted that snakes released at habitat boundaries (AF and U) would orient toward preferred habitat. The snakes from the AF clutch were released at a habitat boundary of agricultural fields and forested wetland; north and east of the clutch site contained agricultural canal systems and south of the clutch site contained herbaceous/woody wetland. We used the V-test to determine whether the initial movement bearings of snakes were significantly oriented toward wetland habitat (south of the clutch site) or canal systems (north/east of the clutch site). The AF clutch was released in close proximity to canals and wetland habitat. We conducted a V-test to determine whether the initial movement bearings of snakes were oriented toward wetland habitat or canal systems (north of the clutch site).

We conducted an additional analysis for the U clutch to determine whether snakes avoided high-intensity urbanized habitat. The U clutch was released in proximity to, but not directly adjacent to, high-intensity urbanized habitat. We investigated habitat boundary behavior in response to high-intensity urbanization using a Monte Carlo approach by generating 1000 random walk paths for each snake using the empirical distribution of step sizes and starting each simulated snake at the release location of the python clutch [42]. Using an alpha of 0.05, we rejected the null hypothesis of no response to habitat boundary if the observed number of habitat crossings by each python fell into the lower 5% of the frequency distribution of number of boundary crossing events from the randomized paths.

## Supplementary Information

**Additional file 1: Supplementary Table 1.** Significance results of Tukey’s range test comparing the mean net movements of snakes from different habitats after 2 months post release. Habitat codes are as follows: forested wetland (FW), agricultural fields (AF), upland pine (UP), and urbanized (U). Note that snake net movement from AF were significantly different from all other habitats.

## Data Availability

The datasets used and analyzed during the current study are available from the corresponding author on reasonable request.

## Abbreviations

CI: Confidence interval
USA: United State of America
km: Kilometers
U: Urbanized Habitat
FW: Forested Wetland
AF: Agricultural Fields
UP: Upland Pine
cm: Centimeter
°C: Degrees Celsius
VHF: Very High Frequency
UTM: Universal Transverse Mercator
NAD83: North American Datum of 1983
GPS: Global Positioning System
g: Grams
ArcGIS: Aeronautical Reconnaissance Coverage Geographic Information System
ANOVA: Analysis of Variance
df: Degrees of freedom
SE: Standard error
n: Sample size
SD: Standard deviation
SVL: Snout to vent length
m: Meters
n/a: Not applicable
