# Supplementary material for: Initial dispersal behavior and survival of non-native juvenile Burmese pythons (Python bivittatus) in South Florida
Source: BMC Zool. 2021 Dec 8;6:33. doi: 10.1186/s40850-021-00098-2 (PMC10124209; doi:10.1186/s40850-021-00098-2)
Supplement: Supplementary file 1 — Additional file 1: Supplementary Table 1. Significance results of Tukey’s range test comparing the mean net movements of snakes from different habitats after 2 months post release. Habitat codes are as follows: forested wetland (FW), agricultural fields (AF), upland pine (UP), and urbanized (U). Note that snake net movement from AF were significantly different from all other habitats. [file 40850_2021_98_MOESM1_ESM.docx]

| Habitat Comparison | Two-Month p-value |
| --- | --- |
| FW - AF | < 0.001 |
| U - AF | <0.001 |
| UP - AF | <0.001 |
| U - FW | 0.998 |
| UP - FW | 0.305 |
| UP - U | 0.273 |

Supplementary Table 1. Significance results of Tukey’s range test comparing the mean net movements of snakes from different habitats after 2 months post release. Habitat codes are as follows: forested wetland (FW), agricultural fields (AF), upland pine (UP), and urbanized (U). Note that snake net movement from AF were significantly different from all other habitats.
